# Dietary Manipulation and Social Isolation Alter Disease Progression in a Murine Model of Coronary Heart Disease

**DOI:** 10.1371/journal.pone.0047965

**Published:** 2012-10-24

**Authors:** Yumiko Nakagawa-Toyama, Songwen Zhang, Monty Krieger

**Affiliations:** Department of Biology, Massachusetts Institute of Technology, Cambridge, Massachusetts, United States of America; University of Tor Vergata, Italy

## Abstract

**Background:**

Mice with a deficiency in the HDL receptor SR-BI and low expression of a modified apolipoprotein E gene (SR-BI KO/ApoeR61^h/h^) called ‘HypoE’ when fed an atherogenic, ‘Paigen’ diet develop occlusive, atherosclerotic coronary arterial disease (CHD), myocardial infarctions (MI), and heart dysfunction and die prematurely (50% mortality ∼40 days after initiation of this diet). Because few murine models share with HypoE mice these cardinal, human-like, features of CHD, HypoE mice represent a novel, small animal, diet-inducible and genetically tractable model for CHD. To better describe the properties of this model, we have explored the effects of varying the composition and timing of administration of atherogenic diets, as well as social isolation vs. group housing, on these animals.

**Methodology/Principal Findings:**

HypoE mice were maintained on a standard lab chow diet (control) until two months of age. Subsequently they received one of three atherogenic diets (Paigen, Paigen without cholate, Western) or control diet for varying times and were housed in groups or singly, and we determined the plasma cholesterol levels, extent of cardiomegaly and/or survival. The rate of disease progression could be reduced by lowering the severity of the atherogenic diet and accelerated by social isolation. Disease could be induced by Paigen diets either containing or free of cholate. We also established conditions under which CHD could be initiated by an atherogenic diet and then subsequently, by replacing this diet with standard lab chow, hypercholesterolemia could be reduced and progression to early death prevented.

**Conclusions/Significance:**

HypoE mice provide a powerful, surgery-free, diet-‘titratable’ small animal model that can be used to study the onset of recovery from occlusive, atherosclerotic CHD and heart failure due to MI. HypoE mice can be used for the analysis of the effects of environment (diet, social isolation) on a variety of features of cardiovascular disease.

## Introduction

Mice with homozygous null mutations in the gene encoding the HDL receptor (scavenger receptor, class B, type I (SR-BI), SR-BI KO) exhibit hypercholesterolemia with abnormally large, unesterified cholesterol-rich HDL particles [1], [2], [3]. When SR-BI KO mice are crossed with mice having homozygous null mutations in the apolipoprotein E (apoE) gene, the progeny SR-BI/apoE double knockout (dKO) mice exhibit severe hypercholesterolemia, dramatically enlarged and abnormal HDL particles and numerous cardinal features of human coronary heart disease (CHD) [4], [5]. SR-BI/apoE double knockout mice (dKO) fed a standard lab chow diet spontaneously develop occlusive coronary atherosclerosis, develop myocardial infarctions (MI), severe heart dysfunction and die young (mean age of 6 weeks). A variant of the dKO, the ‘HypoE’ mouse (SR-BI KO/ApoeR61^h/h^), is a model for diet-induced coronary heart disease [6]. This mouse has homozygous null mutations in the SR-BI gene and a severe, but not absolute, deficiency of apoE due to a modification of the apoE gene (ApoeR61^h/h^). ApoeR61^h/h^ mice developed by Raffai and colleagues [7], [8] express a mutant form of murine apoE, Thr61→Arg61, in place of wild-type (WT) apoE at substantially lower plasma concentrations (2% to 5%) than apoE in control WT mice [6]. Raffai and colleagues have shown that ApoeR61^h/h^ mice (wild-type SR-BI alleles) can develop hypercholesterolemia and aortic atherosclerosis when fed an atherogenic diet and that replacement of the diet with lab chow reduces the hypercholesterolemia and results in regression of the atherosclerosis [7]. We have shown that feeding HypoE mice a relatively harsh, atherogenic diet containing high fat, cholesterol and cholate (Paigen diet: 15.8% fat, 1.25% cholesterol, 0.5% sodium cholate) causes development of extensive atherosclerotic lesions in their aortic sinus and occlusive coronary arterial atherosclerosis, and the mice exhibit profound hypertrophy and cardiac dysfunction secondary to multiple MIs, and die prematurely (∼40 days after initiation of Paigen-diet feeding) [6]. When fed a normal lab chow diet for 3 months, these mice do not develop occlusive atherosclerotic CHD. The HypoE mouse model of CHD has the potential of providing insight into mechanisms of CHD pathophysiology, markers of disease and methods for disease prevention and treatment. If the HypoE mouse is to be a useful tool for the research community, it is important that relevant characteristics of the mouse, e.g., responses to varying diets and husbandry conditions, be well-defined.

Here we have further characterized the HypoE model by exploring the effects of varying the composition and timing of administration of atherogenic diets, as well as social isolation vs. group housing, on the plasma cholesterol levels, cardiomegaly and/or survival. We found that the rate of disease progression could be adjusted by modifying the severity of the atherogenic diet and could be accelerated by social isolation. We also established conditions under which CHD could be initiated by feeding atherogenic diet and then reduction in hypercholesterolemia achieved and progression to early death prevented by replacing this diet with standard lab chow. This protocol may generate a powerful murine model for the study of heart remodeling and heart failure due to MI. Thus, HypoE mice appear to be an attractive model for studying the effects of environment (diet, social isolation) on a variety of features of cardiovascular disease.

## Results

We examined the effects of atherogenic diets with differing severities on the plasma lipids, heart-to-body weight ratios and survival of HypoE mice. The animals were weaned onto a normal chow diet (4.5% fat, 0.022% cholesterol) and at two months of age we began continuous ad lib feeding for the indicated times with one of the four following diets: normal chow, Western diet (21.2% fat, 0.2% cholesterol), Paigen diet without cholate (Paigen NC, 15.8% fat, 1.25% cholesterol), or a standard Paigen diet (15.8% fat, 1.25% cholesterol, 0.5% sodium cholate). Except where indicated otherwise, animals were housed in groups of 4–5 per cage at the beginning of the ad lib feeding of the indicated diets. As indicated below, mortality reduced the population density in some cages.

### Effects of Atherogenic Diets on Plasma Lipids and Lipoproteins

**Figure 1:**
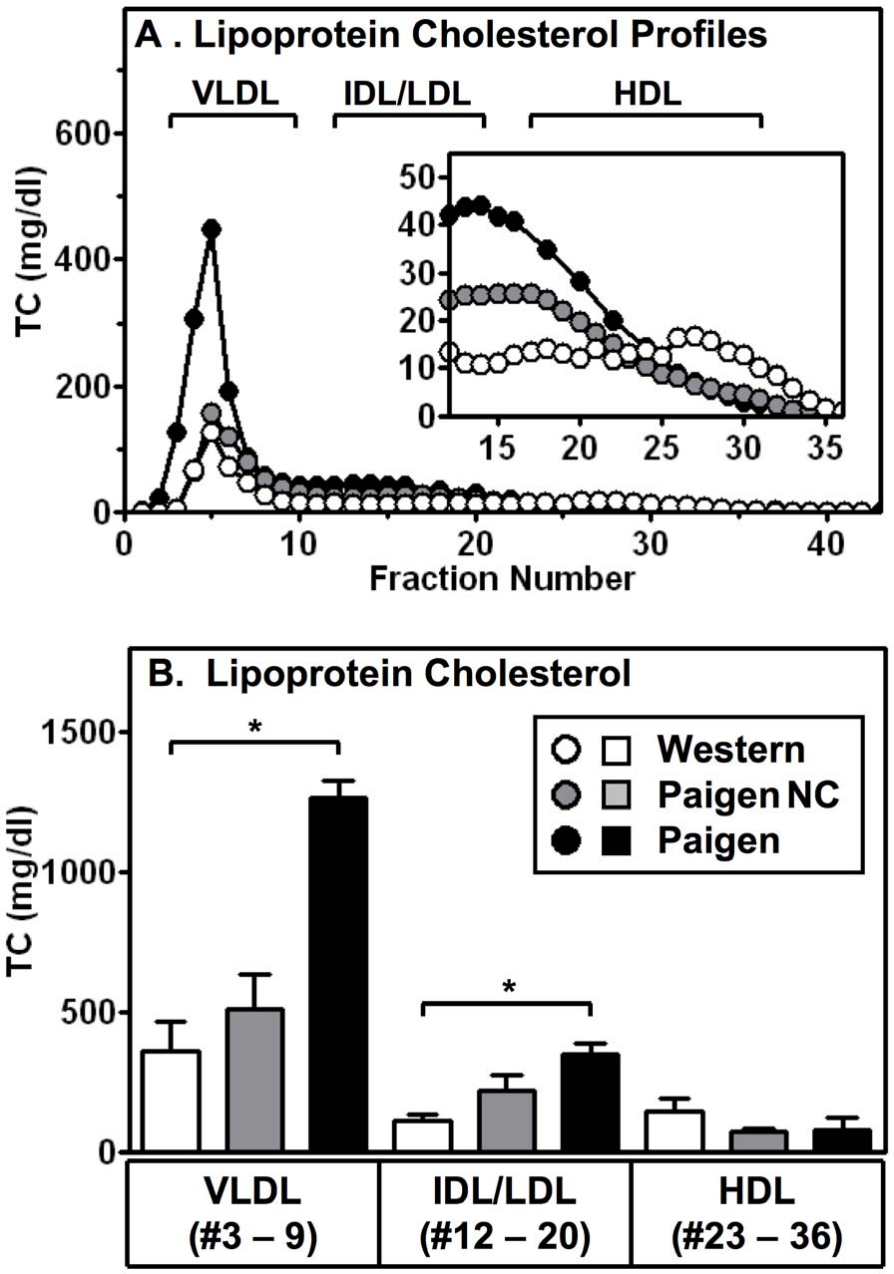
Effects of atherogenic diets on lipoprotein cholesterol profiles of HypoE mice. HypoE mice housed in groups (including one or more SRBI^+/−^ApoeR61^h/h^ littermates per group, 4–5/cage) were fed Western (open/white symbols, n = 5), Paigen NC (gray symbols, n = 5) or Paigen (black symbols, n = 3) diets for one month starting at two months of age and then plasma was harvested and subjected to FPLC size fractionation. The data shown include only the HypoE mice. **A.** Averaged plasma lipoprotein total cholesterol (TC) profiles (mg/dL plasma). Brackets indicate the approximate elution positions of VLDL, intermediate-density lipoproteins (IDL)/LDL, and HDL. **(Inset)** Expanded scale for the IDL/LDL and HDL regions of the profiles. **B.** For each profile from individual mice, total cholesterol levels in the indicated pooled fractions corresponding to VLDL-, IDL/LDL- or HDL-size particles were summed and averages were calculated. Data are represented as mean ± SD. Statistically significant differences were determined by Kruskal-Wallis tests followed by the Dunn’s multiple comparison post-test. *p<0.05.

After administration of one of the four experimental diets for one month, we collected plasma samples and determined both plasma total cholesterol (TC) and unesterified cholesterol (UC) levels (Table 1) and, for the atherogenic diets, lipoprotein particle size distributions (lipoprotein cholesterol profiles determined by FPLC size exclusion chromatography) (Figure 1). Table 1 shows that the TC and UC levels in HypoE mice fed a normal chow diet were significantly higher than those in wild-type mice. Among HypoE mice, the different atherogenic diets had significantly different effects on plasma cholesterol. The differences in the plasma levels of TC, UC or the UC/TC ratios for normal chow and Western diet fed HypoE mice did not reach statistical significance, presumably due in part to large animal-to-animal variations on the Western diet and the multiple hypothesis testing required with four different diets. In contrast, plasma levels of TC and UC were significantly lower on the chow diet than on Paigen NC and Paigen diets. Paigen diet feeding resulted in the highest levels of plasma TC and UC. There were statistically significant differences in these values when the three atherogenic diets were compared as a group (Kruskal-Wallis testing). A statistically significant, higher UC/TC ratio was found in Paigen NC or Paigen diet-fed HypoE mice compared to that in normal chow fed animals, although among the three atherogenic diets there were no significant differences in the UC/TC ratios.

**Table 1:** Effects of atherogenic diets on plasma lipids.

Genotype	Diet	n	TC (mg/dl)	UC (mg/dl)	UC/TC ratio
**WT**	**Normal chow** b (4.5% fat, 0.022% cholesterol)	**5**	**81±31**	**19±9**	**0.23±0.04**
**HypoE** a	**Normal chow** b (4.5% fat, 0.022% cholesterol)	**9**	**282±25** *	**173±19** *	**0.61±0.06** *
	**Western** (21.2% fat, 0.2% cholesterol)	**13**	**704±131** **	**496±144** **	**0.7±0.10**
	**Paigen NC** (15.8% fat, 1.25% cholesterol)	**18**	**918±192**	**692±168**	**0.75±0.06**
	**Paigen** (15.8% fat, 1.25% cholesterol, 0.5%sodium cholate)	**6**	**1630±337**	**1284±274**	**0.79±0.05**

a Mice were housed in groups with mixed genotypes. HypoE mice were fed either a Western diet (n = 13), Paigen diet without cholate (Paigen NC) (n = 18), or Paigen diet (n = 6) for one month beginning at two months of age. Data represent mean ± SD. Statistically significant differences among normal chow and the three atherogenic diets were determined by Kruskal-Wallis tests followed by the Dunn’s multiple comparison post-test.

* p<0.05 vs Paigen NC and Paigen,

** p<0.05 vs Paigen. WT; wild type, TC; total cholesterol, UC; unesterified cholesterol.

b The data for group-housed, wild-type and HypoE mice fed a normal chow diet are from Zhang S, et al (2)-.

Figure 1A shows plasma total cholesterol lipoprotein profiles averaged from multiple HypoE animals fed the Paigen (n = 3), Paigen NC (n = 5) and Western (n = 3) diets. The indicated size ranges for VLDL, ILD/LDL and HDL are for normal plasma lipoproteins. The most striking qualitative patterns seen in the profiles were: 1) in the non-HDL regions of the profiles (fraction #3–22), the relative levels of cholesterol were Paigen>Paigen NC>Western; 2) the VLDL-size peak was much larger for the Paigen diet than the other two diets, and 3) there was a small cholesterol peak in the HDL-size range for Western diet fed mice (see insert, fraction #23–36) not seen in the Paigen NC and Paigen diet fed mice. Figure 1B shows a quantitative analysis of the average TC contents in the standard VLDL- (fractions 3–9), IDL/LDL- (fractions 12–20), or HDL- (fractions 23–36) size ranges. The overall quantitative pattern is consistent with the qualitative analysis; however all of the differences noted did not reach statistical significance. There were statistically significant differences in TC levels in the VLDL-, and IDL/LDL-size ranges between Western and Paigen diets. In contrast, pairwise comparisons of TC levels in the HDL-size range did not show significant differences. Nevertheless, the differences in plasma cholesterol levels and lipoprotein size distributions raised the possibility that the different diets might have different influences on heart disease and survival.

### Effect of Atherogenic Diets on Survival and Cardiac Size

**Figure 2:**
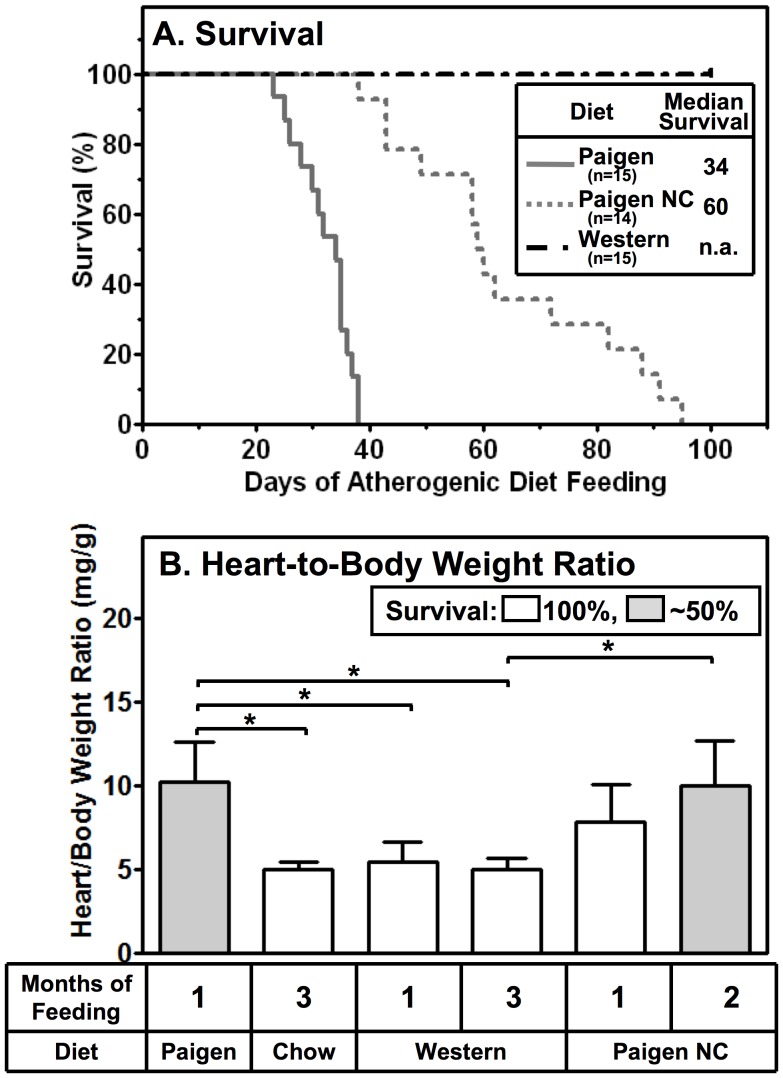
Effects of atherogenic diets on survival (A) and heart-to-body weight ratios (B) of HypoE mice. Mice were housed in groups with mixed genotypes (HypoE and one or more SRBI^+/−^ApoeR61^h/h^ littermates per group, 4–5/cage) and, beginning at two months of age were switched from a normal chow diet to the indicated atherogenic diets. The data shown include only the HypoE mice. **A** Kaplan-Meier survival curves. Mice were fed either Paigen (gray solid line, n = 15), Paigen NC (gray dotted line, n = 14), or Western (black dashed line, n = 15) diets. **B** Heart-to-body weight ratios. For the indicated times (1–3 months), mice were fed the Paigen (n = 14), chow (n = 8), Western (n = 5 (1 month), n = 4 (3 months)), or Paigen NC (n = 6 (1 month), n = 4 (2 months)) diets. Gray and white bars represent those populations in which approximately 50% or 100% of the animals survived after the indicated times of feeding. Data are means ± SD. Statistically significant differences were determined by Kruskal-Wallis tests followed by the Dunn’s multiple comparison post-test. *p<0.05.

Figure 2A shows dramatic differences in the Kaplan-Meier survival curves for HypoE mice (4–5 animals/cage) fed with the different atherogenic diets, beginning at 2 months of age, for up to 100 days. The median survival times after initiating Pagien NC (dashed gray) and Paigen (solid gray) diets were 60 and 34 days, respectively, with no animals surviving for 100 days. In contrast, no animals fed the Western diet died within 100 days after initiating feeding of the atherogenic diets. As previously described for HypoE mice fed a Paigen diet [6], some of the Paigen NC diet fed HypoE mice exhibited gross signs of abnormal heart function prior to death (huddling, shivering/shaking, ruffled fur, and reduced activity).

Because cardiomegaly (about twice the normal heart-to-body weight ratio) is associated with abnormal heart function and MI/premature death in Paigen-diet fed HypoE mice [6], we measured the heart-to-body weight ratios at different times after initiation of the atherogenic diets (Figure 2B). The heart-to-body weight ratio of HypoE mice fed the Paigen diet for one month was twice that of the chow-fed (three months) controls (10.2±2.4 (n = 14) vs 5.0±0.5 (n = 8) mg/g). Western diet feeding for one or three months did not significantly change the heart-to-body weight ratio relative to that for chow-diet fed mice (1 month: 5.5±1.2 mg/g (n = 5); 3 months: 5.1±0.6 mg/g (n = 4)), suggesting that hearts of the Western diet-fed mice, which exhibited no premature mortality, were not dramatically altered. As was the case for mortality (Figure 2A), it seems possible that cardiomegaly in Paigen NC diet-fed HypoE developed more gradually than in Paigen diet-fed animals. After one month of Paigen NC diet feeding, the observed mean heart-to-body weight ratio, 7.8±2.3 mg/g (n = 6), was intermediate between the essentially normal values for either the chow fed controls (3 months) or Western diet-fed mice and the Paigen diet-fed mice (Figure 2B); however there were no statistically significant differences for these comparisons. After two months of Paigen NC diet feeding, by which time half of the mice had died, the heart-to-body weight ratio (10.0±2.7 mg/g (n = 4), gray bar) was significantly higher than those of the chow-fed controls and virtually identical to that of one month (approximate time of 50% survival, gray bar) Paigen-diet fed mice. Thus, the extents of cardiomegaly and the 50% survival times for the different diets appear to correlate and likely reflect different rates of progression of heart disease due to differences in the differing stringencies of the atherogenic diets. As a consequence, varying the atherogenic potency of the diet permits experimental control of the rate of progression of disease in HypoE mice.

### Effect of Social Isolation on the Survival of HypoE Mice Fed Atherogenic Diets

Social isolation increases CHD mortality and morbidity in the general human population and can influence disease in animals as well [9]–[15]. For example, atherosclerosis is higher in singly housed female monkeys than their group housed counterparts [16]–[18]. In our ongoing efforts to assess the value of HypoE mice as a model system for human disease, we compared the effects of social isolation (1 animal/cage) with those of group housing on their survival when fed atherogenic diets.

**Figure 3:**
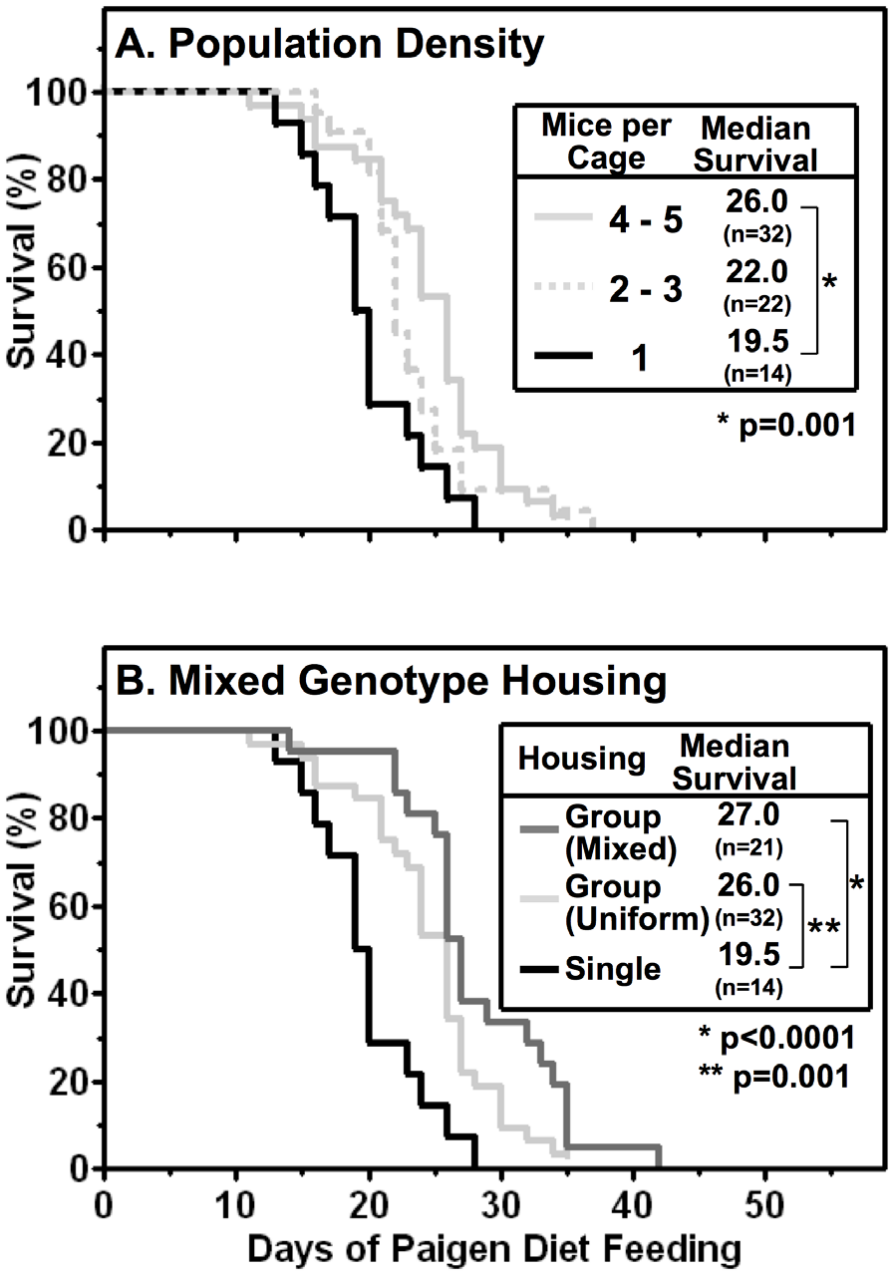
Population density (A) and mixed genotype housing (B) effects on Paigen diet-fed HypoE mouse survival. Mice were housed at the indicated population densities (1, 2–3 or 4–5 (group)/cage) with either uniform (HypoE) or mixed genotypes (HypoE and one or more SRBI^+/−^ApoeR61^h/h^ littermates per group). Beginning at two months of age the animals were switched from a normal chow diet to the standard Paigen diet. The data shown include only the HypoE mice. **A** Kaplan-Meier survival curves for HypoE mice housed singly (1 mouse/cage, black line), or uniform genotype (HypoE only) groups of 2–3 (light gray dashed line), or 4–5 (light gray solid line) mice per cage with the indicated median survival times and number of animals per group (n). A notch in the 4–5 mice per cage line indicates a censored animal that was euthanized due to severe ulcerative dermatitis at 35 days after initiating Paigen diet feeding. **B** Kaplan-Meier survival curves for HypoE mice housed singly (black line) or in uniform genotype (HypoE only, light gray line) or mixed genotype (HypoE and SRBI^+/−^ApoeR61^h/h^ E, gray line) group housing (4–5 mice/cage).

As in the experiments described above, HypoE mice were weaned onto a normal chow diet until two months of age. At two months of age the diets of the mice were switched from chow to an atherogenic diet (Paigen or Paigen NC) and they were placed in cages at population densities of either 1, 2–3, or 4–5 mice per cage (all mice in any one cage were either male or female) with a uniform HypoE genotype. Figure 3A shows Kaplan-Meier survival curves for Paigen diet fed animals. There was no statistically significant difference in the median survival times for animals housed at densities of 4–5 or 2–3 per cage (26 days (n = 32 individuals) vs 22 days (n = 22), respectively). However, there was a strikingly lower median survival time (19.5 days, n = 14) for the singly housed animals compared to those housed at 4–5/cage (p = 0.001). Population density clearly influenced survival kinetics of HypoE mouse fed a Paigen diet.

A potentially confounding feature of the experiment in Figure 3A was that the population density in the group housed (uniform genotype) cages went down as the animals died. We therefore examined the effects of insuring that there was at least one companion mouse present at all times during an experiment for all group-housed HypoE mice by including in each group-housed cage at least 1 littermate whose genotype was SR-BI^+/−^ApoeR61^h/h^ (mixed genotypes). As expected, none of these atherogenic diet-fed companion animals (SR-BI^+/−^ApoeR61^h/h^) died during the experiments reported here. Figure 3B compares the survival curves for uniform genotype group (4–5/cage, light gray line) and singly (1/cage, black line) housed mice (same data as in Figure 3A) with that of mixed genotype group housed mice (4–5/cage, dark gray line). Median-survival time for the HypoE mice in the mixed genotype group (27.0 days, n = 21) was not significantly longer than for the uniform genotype group (26.0 days, p = 0.053), but was significantly longer than for the singly housed animals (p<0.001), indicating that there was no clear advantage to using the mixed genotype housing protocol with Paigen diet feeding. In the experiments described below, the group housed animals included either uniform or mixed genotypes, as indicated in the Figure legends. For those cases in which mixed genotype, group housing was used, results are reported only for the HypoE mice (not the companion, SR-BI^+/−^ApoeR61^h/h^ mice).

**Figure 4:**
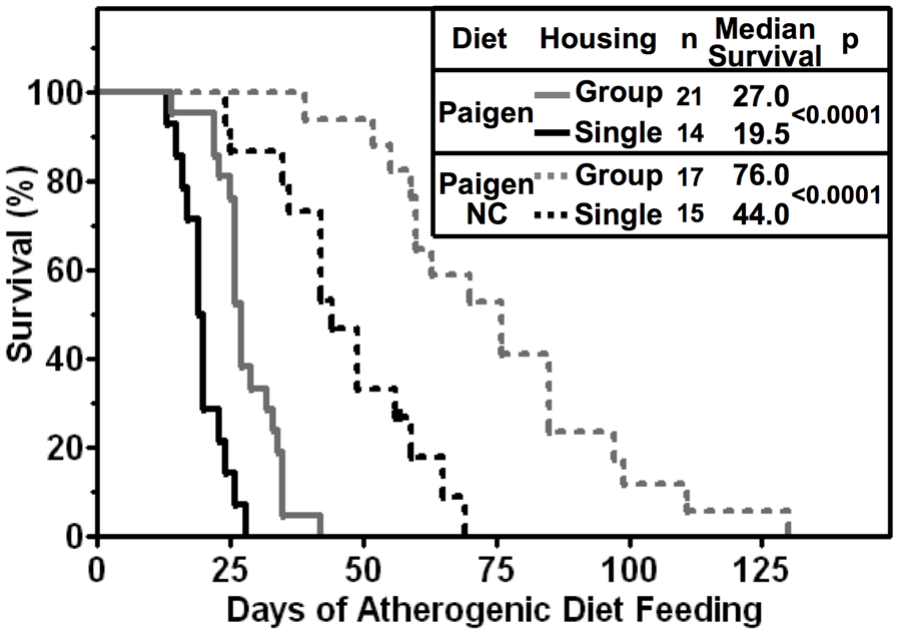
Effects of population density and atherogenic diets on the survival of Paigen diet-fed HypoE mice. HypoE mice were housed singly (1/cage, black lines) or in mixed genotype groups (HypoE and one or more SRBI^+/−^ApoeR61^h/h^ littermates per group, 4–5/cage, gray lines). Beginning at two months of age the animals were switched from a normal chow diet to either the standard Paigen (solid lines) or Paigen NC (dashed lines) diets. The Kaplan-Meier survival curves shown include only the HypoE mice. A notch in the singly housed HypoE mice fed the Paigen NC diet indicates a censored animal, which was euthanized due to severe ulcerative dermatitis at 57 days after initiating Paigen NC diet feeding.

Figure 4 compares the population density effect on HypoE survival for mice fed the Paigen diet (solid lines) with that for mice fed the less severe Paigen NC diet (dashed lines). As was the case with the Paigen diet, singly housed mice fed the Paigen NC diet exhibited a shorter median survival time (44.0 days, n = 15) than mixed genotype group housed HypoE mice (76.0 days, n = 17, p<0.0001). Thus, the population density effect was not dependent on the cholate present in the Paigen, but absent from the Paigen NC, diet.

The data in Figures 3 and 4 were pooled from experiments using both male and female mice. Table 2 shows median survival data stratified by sex. For each sex and diet (Paigen, Paigen NC), the median survival times were significantly lower (25–30%) for single than the corresponding group housing. In the case of the Paigen diet, there were no significant differences in median survival between males and females. For Paigen NC-diet feeding, the median survival time for both males and females was ∼33% shorter than for group housing. The absolute difference in survival time between group housed, Paigen NC-diet fed males and females (85 vs 63 days) was not statistically significant; however, the difference between singly housed males and females (57.5 vs 42 days) was significant (p = 0.002). Overall, the effects of population density and diet on survival of HypoE mice seen for the pooled data were also observed when males and females were analyzed independently.

**Table 2:** Effects of Atherogenic Diets, Population Density and Sex on Survival.

Diet^a^	Housing	Sex	n	Median Survival (Days)^b^	p
Paigen	Group	Male	11	26.0^#^	0.99
		Female	10	27.0*	
	Single	Male	9	20.0^#^	0.14
		Female	5	19.0*	
Paigen NC	Group	Male	8	85.0^##^	0.46
		Female	9	63.0**	
	Single	Male	8	57.5^##^	0.002
		Female	7	42.0**	

a From two months of age HypoE mice of the indicated sexes were fed either the Paigen or Paigen NC diets and housed either singly (one/cage) or in groups of 4–5 mice per cage, with the groups containing HypoE and one or more SRBI^+/−^ApoeR61^h/h^ littermates. The values of median survival are for HypoE mice only.

b *, **p<0.0001 ^#^, ^# #^p<0.02.

To determine if more rapid onset of cardiomegaly was associated with the shorter median survival times observed in singly housed mice, we measured the heart-to-body weight ratios of singly and group housed male and female HypoE mice fed the Paigen diet for 19 days (median survival time for single housing). Indeed, the heart-to-body weight ratios were higher in the singly housed mice. The ratios (mg/g) for males were: group housing, 6.9±0.2 (n = 19); single housing, 11.0±0.5 (n = 16) (p<0.0001) and for females: group housing, 8.3±0.4 (n = 24); single housing 11.2±0.5 (n = 13) (p<0.0001). Thus, it seems likely that more rapid onset of CHD in the singly housed mice was responsible for the shorter median survival times.

### Effect of Social Isolation on Plasma Cholesterol Levels of HypoE Mice Fed Atherogenic Diets

**Figure 5:**
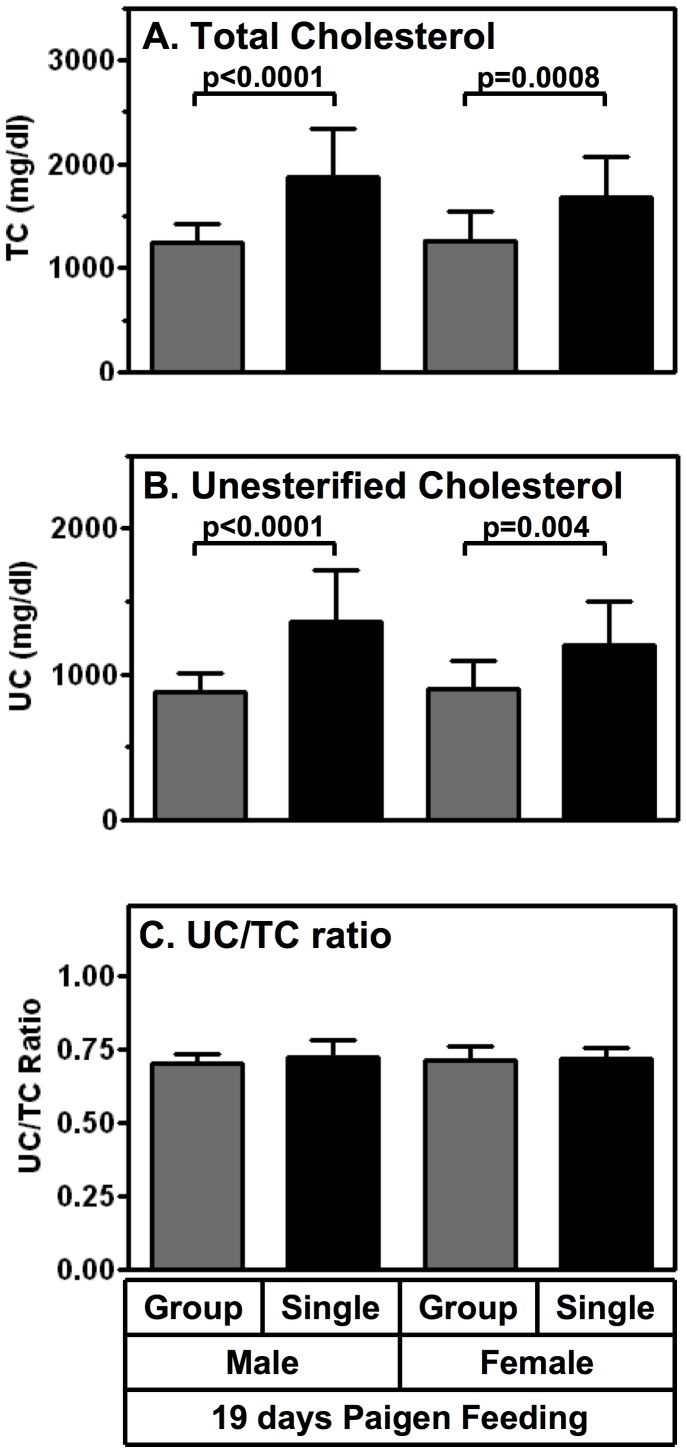
Effects of population density and sex on plasma cholesterol levels of Paigen diet-fed HypoE mice. HypoE mice were housed singly (1/cage, black bars; males, n = 16, females; n = 13) or in mixed genotype groups (HypoE and one or more SRBI^+/−^ApoeR61^h/h^ littermates per group, 4–5/cage, gray bars; males, n = 19, females; n = 24). Beginning at two months of age the animals were switched from a normal chow diet to the Paigen diet. After 19 days of Paigen diet feeding, plasma was harvested and total cholesterol (TC), unesterified cholesterol (UC) and UC/TC ratios were determined from the HypoE mice. Data are means ± SD. Statistically significant differences were determined by unpaired Student’s t or Mann-Whitney test.

The principle goal of the current studies was to describe the characteristics of HypoE mice. However, we have begun to explore potential mechanisms underlying the differences in the rate of development of cardiomegaly and median survival times of singly and group housed HypoE mice fed the Paigen diet. First, we measured the cholesterol (TC and UC) concentrations determined from plasma sampled at two times after initiation of the atherogenic diet: 19 days (∼median survival time of singly housed HypoE mice) and 10 days (prior to any deaths or evidence of reduction in weight gain or loss of weight associated with severe CHD). Figure 5 shows that after 19 days on the Paigen diet there were significantly higher plasma levels of TC (32–50%, panel A) and UC (34–55%, panel B) in both male and female singly housed mice compared to their group housed counterparts. There were no significant differences in the UC/TC ratios (panel C) between the different sexes or population densities.

The results were similar for male HypoE mice fed the Paigen diet for only 10 days. Compared to group housed animals, singly housed male mice exhibited 41% higher TC (1602±89 vs 1133±56 mg/dl; p = 0.002) and 39% higher UC (1036±66 vs 743±40; (p = 0.002), n = 6/set). There were no significant differences in the UC/TC ratios (group: 0.65±0.01, single: 0.65±0.01 p = 0.59). Thus, the population density effect on plasma cholesterol preceded any gross, outward signs of pathology and may have contributed to the dependence of the rate of disease progression on population density.

### Exploration of Potential Sources of the Population Dependent Differences in Plasma Cholesterol Levels

One potential explanation for the differences in plasma cholesterol levels in Paigen-diet fed single versus group housed mice is differences in food intake. We therefore measured apparent daily food intake, defined as the difference in unconsumed food weight in the food reservoirs of the cages between sequential days. Female HypoE mice exhibited an unexpected eating behavior not shared by their male counterparts or by females with other genotypes that were examined (WT, SR-BI^+/−^ApoeR61^h/h^, or SR-BI^−/−^ mice with wild-type apoE genes). They crumbled the food pellets, generating substantial amounts of crumbs in the bedding of the cage that were difficult to quantify reliably with our experimental apparatus (see Figure S1). We do not know why these mice exhibited this unexpected behavior. Because of it, however, we limited our analysis to male mice.

Figure S2A shows that there were no obvious differences in the apparent daily food intake per mouse from days 2 to 10 after initiation of Paigen diet feeding for singly housed HypoE mice, singly housed controls (WT and SR-BI^+/−^ApoeR61^h/h^, combined data), or group housed mice with mixed genotype (HypoE and SR-BI^+/−^ApoeR61^h/h^), even though there were differences in plasma cholesterol levels at 10 days between singly and group housed HypoE mice (see above). Figure S2B shows the mean daily food intake averaged over days 2–10. There were no statistically significant differences in the mean daily food intake values (g/day/mouse, p = 0.70): Group/mixed genotype, 3.6±0.4 (n = 6 HypoE mice,13 total mice ); Single/HypoE, 3.9±0.8 (n = 6); and Single/control, 3.9±0.1 (n = 6). These observations suggest that there was not a significant difference in atherogenic diet intake between singly and group housed HypoE mice. This conclusion is consistent with our observation that the body weights of the group and singly housed HypoE mice determined 10 days after initiation of the Paigen diet feeding were not significantly different (26.2±4.0 g and 25.2±2.1, respectively; p = 0.31, n = 6/set). Thus differences in the amounts of Paigen diet ingested are unlikely to account for the differences in the plasma TC and UC levels in singly compared with group housed HypoE mice.

**Figure 6:**
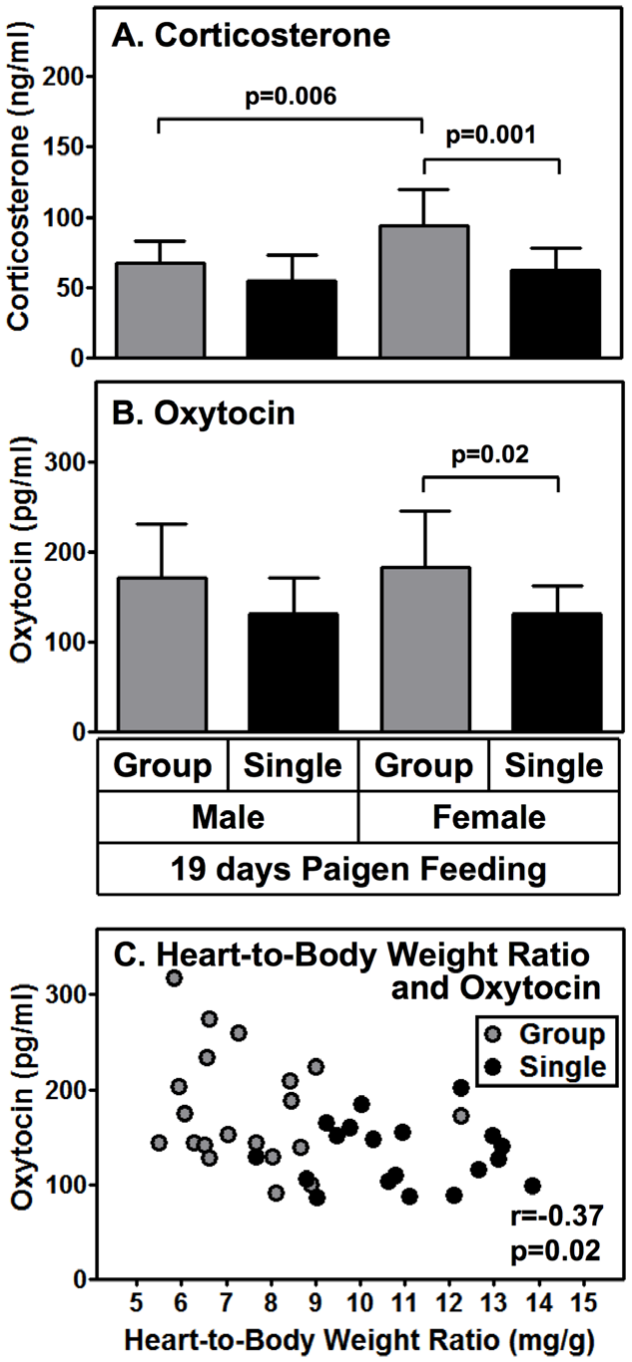
Population density and sex effects on plasma corticosterone and oxytocin in Paigen diet-fed HypoE mice. HypoE mice were housed in mixed genotype groups (HypoE and one or more SRBI^+/−^ApoeR61^h/h^ littermates per group, 4–5/cage, gray bars) or singly (1/cage, black bars). Beginning at two months of age the animals were switched from a normal chow diet to the Paigen diet. After 19 days of Paigen diet feeding, the mice were weighed, plasma and hearts were harvested, heart weights measured and plasma levels of corticosterone and oxytocin were determined. The values presented are from the HypoE mice only; they do not include the companion (group housing) SR-BI^+/−^ApoeR61^h/h^ mice. **A** Plasma corticosterone levels: group housed males, n = 11; singly housed males, n = 14; group housed females, n = 16; singly housed females, n = 12. **B** Plasma oxytocin levels: group housed males, n = 8; singly housed males, n = 8; group housed females, n = 12; singly housed females, n = 11. Data are means ± SD. Statistically significant differences were determined by unpaired t-test. **C** Correlation of oxytocin levels and heart-to-body weight ratios for all HypoE mice (n = 39). Gray and black circles indicate group and single housing, respectively. Statistics were evaluated using Spearman’s rank correlation.

Differences in the endocrine systems (e.g., stress) [19], [20] is another potential source of population density dependent differences in plasma cholesterol levels, the rates of development of cardiomegaly and median survival times. In mice, social isolation can decrease body weight gain and food consumption, increase stereotypic and vertical movements, basal corticosterone levels and aggressiveness relative to group-housed animals [21]. Therefore, 19 days after initiating Paigen-diet feeding, we determined plasma levels of corticosterone and oxytocin in male and female HypoE mice. Corticosterone is a well known stress marker and oxytocin regulates social behavior [22] and was reported to influence the cardiovascular system [23]. Figure 6A shows that there were no statistically significant differences in plasma corticosterone levels between singly and group housed males (n = 11 or 14, p = 0.08). The absence of an effect of isolation on plasma corticosterone in HypoE mice was not surprising in the light of the previous studies of stress in male and female SR-BI KO mice showing their markedly reduced corticosterone response to stress (fasting, LPS and bacterial infection, ACTH, cold-water swimming) [24], [25]. Thus, differences in corticosterone levels in males cannot account for the striking population density differences in plasma cholesterol and survival we have observed in males. We did note that the corticosterone level in group housed females was higher than those in either group housed males or singly housed females. We did not observe any common trends in the relationships of plasma corticosterone levels and heart-to-body weight ratios when we compared separate analyses of male and female mice. The mechanism underlying the sexual dimorphism seen here is unclear, although sexual dimorphism in the murine hypothalamic-pituitary-adrenal axis has been reported [26].

Plasma oxytocin levels (Figure 6B) in female HypoE mice fed the Paigen diet for 19 days were significantly lower for single versus group housing (group: 183±62 pg/ml; single: 132±30 pg/ml; n = 12–11, p = 0.02). However a similar difference seen in males (group: 172±59; single: 132±40; n = 8/group) did not reach statistical significance (p = 0.14) (Figure 6B). Figure 6C shows that there was a significant inverse relationship between plasma oxytocin levels and heart-to-body weight ratios when all of the data were pooled (n = 39; r = −0.37p = 0.02). It is not clear if this inverse association reflects a causal relationship or independent coincidence.

### Effects of Withdrawing the Paigen Diet on the Survival of HypoE Mice and their Plasma Cholesterol Levels

The ability to induce fatal CHD in HypoE mice by administering an atherogenic diet (e.g., the Paigen diet) raised the possibility that exposure to this diet for only a relatively brief period followed by feeding a normal chow diet might be sufficient to induce CHD (e.g., cardiomegaly) but not premature death. Such conditions might permit the HypoE mice to be used as a model for cardiac remodeling and recovery from atherosclerosis associated heart damage. Therefore, we initiated Paigen diet feeding of singly housed mice at two months of age as described above, but subsequently replaced the Paigen diet with a normal chow diet 10, 12 or 14 days later, or continued to feed the Paigen diet for the remainder of the study (control).

**Figure 7:**
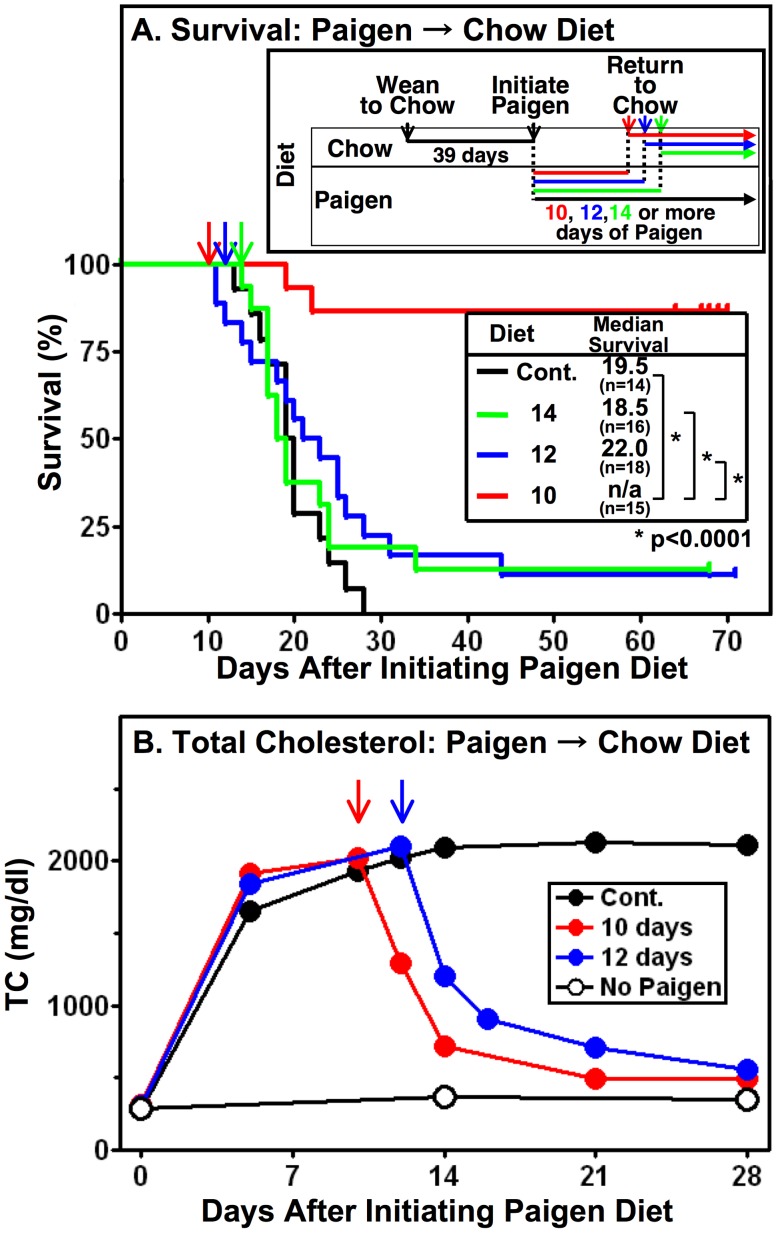
Effects of shifting from Paigen to chow diet on survival (A) and plasma cholesterol (B). HypoE mice were fed a normal chow diet) from weaning to 2 months of age (39 days). Beginning at two months of age the mice were housed singly (1/cage, both males and females were included) and some of the animals were continued to be fed the chow diet (panel B, white circles) whereas all of the others where switched from a normal chow diet to the Paigen diet (initiation of Paigen diet). The animals were maintained continuously throughout the rest of the experiment on the Paigen diet (“Cont.”, black lines) or after the indicated periods of Paigen diet feeding (10 days, red; 12 days, blue; 14 days, green) the mice were returned to a chow diet for the remainder of the experiment (colored arrows), as indicated in the schematic inset in panel A. **A** Kaplan-Meier survival curves. Log-rank test was performed to compare survival curves. **B** Time course of changes in plasma total cholesterol levels. Blood samples were taken serially at the indicated times (see Methods) from subgroups of animals different from those used for the survival curves in panel A (Cont.;n = 9, 10 days; n = 6, 12 days; n = 9, No Paigen(chow); n = 6). The data are presented as mean values of TC in each subgroup at the indicated times. In those cases in which animals died during the period of serial sampling, the data obtained from those mice while alive were incorporated into the calculation of the mean values.

Figure 7A shows Kaplan-Meier survival curves from this experiment. As expected, all of the control mice continuously fed the Paigen diet (black line) died by 30 days with a median survival time of 19.5 days. The survival curves were not dramatically altered if the Paigen diet was administered for only 12 (blue line) or 14 (orange line) days, although several animals survived longer than 30 days. Remarkably, if Paigen diet feeding was limited to only 10 days (red line), most of the animals (87%) survived for at least an additional 60 days.

The heart-to-body weight ratio 10 days after initiation of the Paigen diet feeding was 8.9±1.2 (n = 5), 1.8–times larger than for chow diet fed animals (∼5.0), indicating substantial cardiomegaly. Cardiomegaly (heart-to-body weight ratio 8.2±1.4 (n = 13)) in 10 day transiently Paigen-diet fed mice also was observed after an additional 60 days of chow feeding. Although substantial, these heart-to-body weight ratios were significantly smaller than that observed in mice continuously fed the Paigen diet for 19 days (11.1±1.8 (n = 29), p<0.05), indicating that an upper limit of cardiomegaly had not been reached. There was a very sharp temporal threshold of approximately 10 days of Paigen diet feeding, two days beyond which the progression to death could not readily be prevented simply by withdrawal of the atherogenic diet. Time-course analysis of plasma TC levels revealed that there were no obvious differences between 10 and 12 days of Paigen diet feeding on the maximal plasma TC level attained nor in the shape of the TC decay curves after withdrawal from the Paigen diet (Figure 7B). It seems likely that the two additional days of exposure to the diet were sufficient to tip the balance from primarily long term survival to nearly inevitable death.

## Discussion

In the current studies, we provide additional phenotypic characterization of HypoE (SR-BI KO/ApoeR61^h/h^) mice that represent a novel, diet-induced model of atherosclerotic CHD. We have explored the effects of varying the composition and timing of administration of atherogenic diets, as well as social isolation vs. group housing, on the plasma cholesterol levels, cardiomegaly and/or survival of HypoE mice. These mice do not express the HDL receptor SR-BI and they express no endogenous apoE and only very low levels of a human E4-like isoform of murine apoE (Thr61→Arg61). The Thr61→Arg61 substitution was initially designed to convert the murine apoE from a protein expected to resemble the human E3 isoform of apoE to one more like that of the human E4 isoform [7], [8], [27]. In vivo the replacement of wild-type murine apoE with normal levels of the mutant Thr61→Arg61 protein does not significantly alter plasma total cholesterol or triglyceride levels nor does it change the lipid distribution of plasma lipoproteins; although as expected the Thr61→Arg61 protein exhibited a preference for association with larger lipoproteins relative to the wild type murine apoE in Arg61/wt heterozygotes [27].

The diets used here included a control, normal chow diet (4.5% fat, 0.022% cholesterol) and atherogenic diets of increasing severity: Western diet (21.2% fat, 0.2% cholesterol), Paigen diet without cholate (Paigen NC, 15.8% fat, 1.25% cholesterol), and a standard Paigen diet (15.8% fat, 1.25% cholesterol, 0.5% sodium cholate). The most sever atherogentic diet (Paigen) caused the greatest increase in plasma cholesterol carried in large (VLDL- and IDL-size) lipoprotein particles, most rapid development of cardiomegaly, and shortest survival times (medial survival on the diet of 34 days). The intermediate severity diet (Paigen NC) induced a less dramatic increase in large lipoproteins, a more gradual onset of cardiomegaly and somewhat longer survival times (median survival of 60 days). Thus, as in previous studies (6,32), the current study shows that the extent of cardiomegaly is correlated with the median age of death. The results using the Paigen NC diet show that disease development in HypoE mice did not require cholate, whose inclusion in atherogenic diets is associated with hepatic fibrosis/inflammation [28] formation of macrophage-derived,multinucleated giant cells in atherosclerotic plaque [29]. Getz and Reardon have reviewed the use of varying atherogenic diets, including the influence of cholate, in studies of murine atherosclerosis [30]. When HypoE mice were fed the relatively mild Western diet, they exhibited no significant cardiomegaly after 3 months of feeding nor impaired survival (observed for 100 days) compared to control, chow fed animals. It is possible that the significantly higher levels of dietary cholesterol in the Paigen and Paigen NC diets (1.25%) than in the Western diet (0.2%) may have dramatically influenced cardiac disease development in HypoE mice. Additional studies will be required to precisely delineate the mechanisms underlying the diet-dependent differences in cardiac disease development. The results described here establish that the severity of disease in HypoE mice can be controlled by varying the severity of the atherogenic diet.

The rapid onset and progression of atherosclerosis, CHD, and early death in hypercholesterolemic SR-BI/apoE dKO mice fed a normal chow diet [4], [5] and HypoE mice fed a standard Paigen [6] or Paigen NC (this study) diet can be advantageous for a variety of studies of atherosclerotic CHD. For example, these models of CHD are distinguished by the short duration of experiments, ability to sensitively detect genetic or pharmacologic changes in disease onset and progression in relatively small numbers of animals because of the very steep survival curves and absence of reliance on surgical interventions [2], [4], [31]–[33]. However, in some cases the highly rapid onset and progression of the aggressive occlusive atherosclerosis may limit the usefulness of dKO and Paigen-diet fed HypoE mice in studying important features of CHD and potential therapies. These models may not be ideal for studying collateral vessel formation (therapeutic angiogenesis), the effects of slowly developing cardiac remodeling due to MI and consequent heart failure, preconditioning, and certain dietary, genetic, and pharmacological therapies that may be insufficiently potent to arrest or reverse the highly aggressive disease, but may be effective in milder disease that may better model some features of typical human CHD. We have previously shown that we can slow the kinetics of fatality in SR-BI/apoE dKO mice by varying the timing of administration and/or withdrawal of probucol, a hypolipidemic and antioxidant drug that dramatically slows the onset and progression of CHD in these mice [2]. However, the effectiveness of probucol may be limited by uncertainty about the mechanism underlying probucol’s dramatic effects, potential limitations in controlling these effects (probucol is a relatively hydrophobic drug that has the potential of accumulating in adipose tissue) and our observation that extended treatment with probucol can result in unexpected cardiac phenotypes in SR-BI/apoE dKO mice (in preparation). Therefore, the ability to ‘tune’ disease progression in HypoE animals by dietary manipulation as established in the current studies may prove useful for studies of CHD in which highly rapid onset and aggressive occlusive atherosclerosis with early death is not desirable.

### Social Isolation Shortened the Lifespan of HypoE Fed with Paigen and Paigen NC Diets

The deleterious effects on health of stress and social isolation have been recognized for decades [34]–[36]. Social isolation increases CHD mortality and morbidity in the general human population and can influence disease in animals as well [9]–[14]. For example, atherosclerosis is higher in singly housed female monkeys than their group housed counterparts [15]–[17]. In mice, social isolation (1 animal per cage) can decrease body weight gain and food consumption, increase stereotypic and vertical movements, basal corticosterone levels and aggressiveness relative to group-housed animals [21]. Bemberg et al. have reported that social isolation of apoE KO mice increases their plasma lipids and atherosclerosis in their innominante arteries [20]. In contrast, Lin et al have reported that increased population density results in increased plasma lipids and increased atherosclerosis lesion severity in wild-type C57/BL6 mice [37].

Although we have seen no differences in the survival of SR-BI/apoE dKO mice housed singly or in groups, we observed a profound reduction in longevity in singly housed HypoE mice when fed either a Paigen- (by 7 days) or Paigen NC- (by 32 days) diet. Strikingly, although social isolation did not appear to affect food intake or body weight, it resulted in significantly higher plasma TC and UC levels during a 10-day course of Paigen diet feeding. It seems likely that the increased exposure to higher plasma cholesterol levels could promote more rapid atherosclerosis development and thus shorter survival. We have observed that the qualitative nature of the occlusive atheroscleorosis and MI at the median age of death for singly housed HypoE mice are similar to those seen at the median age of death for the group housed mice (unpublished), indicating that occlusive coronary arterial atherosclerosis is the likely pathophysiological mechanism responsible for the premature death.

To explore the potential relationship of alterations in the endocrine system with the effects of social isolation, we measured plasma levels of two hormones: corticosterone and oxytocin. Corticosterone is an adrenocortical steroid whose plasma levels are often used as a quantitative measure of stress, although SR-BI KO mice exhibit defective plasma corticosterone responses to stress [24], [25]. Thus, plasma corticosterone levels cannot be used as a reliable indication of stress in SR-BI KO mice. We observed no difference in plasma corticosterone levels in group and singly housed male HypoE mice. Nor did we observe a consistent correlation between plasma corticosterone levels and heart-to-body weight ratios when males and females were analyzed separately. Thus, differences in corticosterone levels, elevations in which were seen in group housed females, are unlikely to account for the effects of social isolation on plasma cholesterol and survival.

Oxytocin, a hypothalamic neuropeptide produced in the posterior pituitary gland and other tissues (including the heart), can regulate behavioral responses to stressors and the reactivity of the hypothalamic-pituitary-adrenal (HPA) axis [38], [39].There are reports of oxytocin-mediated regulation of cardiovasucular activity [23], [40]. There is also a report of sexual dimorphism in plasma oxytocin response (elevation in females, but not males) to social isolation in prairie voles [41]. Thus, oxytocin may link social isolation and cardiovascular disease. Plasma oxytocin levels in socially isolated HypoE mice was lower than in group housed controls, but the difference only reached significance in females when males and females were analyzed separately. Strikingly, there was a significant, inverse correlation between plasma oxytocin level and heart-to-body weight ratio (an indicator of heart disease) when all HypoE mice (male, female, group and singly housed) were included in the analysis. These findings are consistent with the report of Ondrejcakova et al. that oxytocin can be cardioprotective in a rat model of ischemia-reperfusion-induced myocardial injury [42]. We found no correlation between plasma corticosterone and oxytocin levels in Paigen diet-fed HypoE mice (r = 0.27, p = 0.14; Figure S3). This may be a consequence of the inability of SR-BI KO mice to respond to stress by increasing corticosterone output [24], [25]. Additional analysis of the mechanisms underlying the effects of social isolation on disease progression in atherogenic diet-fed HypoE mice, which are likely to be complex and multifaceted, may help clarify some of the mechanisms responsible for the well-established psychosocial influences on health in general and cardiovascular disease in particular.

### Paigen Diet Withdrawal Extended HypoE Mice Survival

A particularly intriguing feature of the HypoE mouse is the potential of initiating the development of CHD by feeding an atherogenic diet and then replacing that diet with a normal chow diet to stop additional atherosclerosis progression. Indeed, we found that the hypercholesterolemia in HypoE mice that reaches a steady state after five days of Paigen diet feeding can be substantially reversed after returning the mice to a chow diet for four days. When HypoE mice were fed the Paigen diet for 10 days, which causes profound cardiomegaly (and presumably cardiac damage due to MI) and then returned thereafter to a chow diet, most of the animals (87%) were able to survive for at least 60 days. At that time, the mice exhibited cardiomegaly and preliminary histology studies raise the possibility that their hearts may have undergone remodeling during the 60 day post-atherogenic diet period. Thus, HypoE mice might be a promising novel model for the study of heart remodeling, reversion of occlusive coronary arterial atherosclerosis (also see 3), and the pathophysiology of heart failure without the need for complicating surgical procedures such as coronary ligation.

## Materials and Methods

### Animals and Diets

All mice were on mixed C57BL/6x129 backgrounds (50/50:WT, SR-BI KO, and HypoE) [6] and were maintained under standard light (12 hr, on at 7 am)/dark (12 hr) conditions in the animal facilities at the Massachusetts Institute of Technology. Genotypes were determined by polymerase chain reaction. As anticipated from previous studies of SR-BI KO mice, female, but not male, HypoE (SR-BI KO/ApoeR61^h/h^) mice are infertile [5], [6]. Thus, female ApoeR61^h/h^ mice with heterozygous null mutations in SR-BI were mated to generate the HypoE mice. Mice were fed a normal chow (low fat) diet (4.5% fat, 0.022% cholesterol, Prolab3000, PMI Feeds) from weaning to 2 months of age. Then they were fed either Western (21.2% fat, 0.2% cholesterol, TD 88137, Harlan-Teklad, Madison, WI), Paigen without cholate (Paigen NC) (15.8% fat, 1.25% cholesterol, TD94059, Harlan-Teklad, WI), or Paigen (15.8% fat, 1.25% cholesterol, 0.5% sodium cholate, TD 88051, Harlan-Teklad, WI) diets for the indicated periods. In some cases animals fed the Paigen diet were switched back to a standard chow diet, as indicated. After weaning (3 weeks of age) mice were housed with the same sex littermates until 2 months of age. Animals from multiple, independent litters were distributed among the different experimental groups. Number of animals used in each experiment are indicated (‘n’).

#### Ethics statement

All experiments using animals described here were performed in strict accordance with NIH and Massachusetts Institute of Technology guidelines and approval of the Committee on Animal Care at the Massachusetts Institute of Technology (approved protocol numbers 0209-015 and 0212-015).

### Gravimetry

Mice were anesthetized by intraperitoneal injection of 1.25% Avertin, weighed, and perfused with heparinized PBS and intact hearts were dissected and weighed.

### Determination of Plasma Lipids and Lipoprotein Cholesterol Content

Levels of plasma total cholesterol (TC) and unesterified cholesterol (UC) were determined using standard assay kits from Wako Chemicals (Richmond, VA), according to the vendor’s instructions. FPLC plasma fractionation was performed as previously described [2].

### Population Density and Mixed Genotype Housing

The experimental housing conditions from 2 months of age were as follows: (a) “group housing”: mice with the same genotype (e.g., HypoE) and sex were housed in groups of 2 to 5 per cage, (b) “group housing with mixed genotype”: mice of the same sex but different genotypes (HypoE and SRBI^+/−^ApoeR61^h/h^) were housed in groups of 4 to 5 per cage, and (c) “isolated” or “single” housing: mice were individually housed in cages. All of the cages were equal in size.

### Determination of Food intake

Apparent daily food intake (defined as the difference in unconsumed food weight between sequential days) was recorded daily from day 2 to day10 after initiation of Paigen diet feeding of singly housed mice (HypoE, WT or SR-BI^+/−^ApoeR61^h/h^) and group housed mice with mixed genotypes (HypoE with SRBI^+/−^ApoeR61^h/h^). For group housed mice, the intake per mouse is the total divided by the number of mice in the cage (n = 4 or 5) and thus represents an average contributed to by mice with both genotypes.

### Determination of Plasma Hormone Levels

Plasma was isolated from blood drawn between 10∶00 am and 12∶00 pm and samples were stored at −80°C. Plasma oxytocin and corticosterone levels were determined in thawed samples using a commercially available enzyme immunoassay (Assay Designs, Ann Arbor, MI). The genotypes of mice used for plasma hormone measurements were all HypoE, and group housed SR-BI^+/−^ApoeR61^h/h^ mice were not included in the group housed cages for these measurements.

### Paigen Diet Withdrawal Study

Singly housed HypoE mice were fed the Paigen diet for 10, 12, or 14 days from the age of 2 months, then were fed a normal chow diet thereafter. For some animals, small amounts of blood were drawn serially from the retro-orbital plexus with heparinized capillary tubes for determination of plasma TC after 0, 5, 10, 12, 14, 16, 21 or 28 days after initiating feeding the Paigen diet, as indicated. Controls included mice fed only a chow diet throughout the experiment or those fed a Paigen diet without changing back to a chow diet.

### Statistical Analysis

Data are shown as the means ± S.D. Statistically significant differences were determined by Kruskal-Wallis tests followed by the Dunn’s multiple comparison post-test for comparison of three or more groups, two-tailed, unpaired Student’s t or Mann-Whitney test for comparison of two samples, and log-rank test to compare survival curves. Correlation between heart-to-body weight ratio and plasma hormone levels was evaluated by Pearson’s correlation or Spearman’s rank correlation. A value of *p*<0.05 was considered significant.

## Supporting Information

Figure S1 **Effects of population density on apparent daily food intake of Paigen diet-fed HypoE female mice.** Female mice were housed in mixed genotype groups (4 mice/cage, 2 HypoE and 2 SRBI^+/−^ApoeR61^h/h^ littermates per group, 3 cages, gray symbols, n = 6 for HypoE mice) or singly (1/cage, black symbols; genotypes: HypoE, solid line, n = 5; wild-type (WT) or SRBI^+/−^ApoeR61^h/h^ mice, dashed line, n = 4). Beginning at two months of age the animals were switched from a normal chow diet to the Paigen diet and then the food reservoir was weighed daily for each cage. The estimated food intake per day per mouse was calculated as the difference between reservoir weights on sequential days. The per mouse values for the group housed, mixed genotypes are averages from all of the animals in each cage.
(TIF)

Figure S2 **Effects of population density on apparent daily food intake of Paigen diet-fed HypoE male mice.** Male mice were housed in mixed genotype groups (HypoE and one or more SRBI^+/−^ApoeR61^h/h^ littermates per group, 3 cages, 4–5 mice/cage, 1–3 HypoE mice/cage, gray symbols, n = 6 for HypoE mice) or singly (1/cage, black symbols; genotypes: HypoE, solid line, n = 6; wild-type (WT) or SRBI^+/−^ApoeR61^h/h^ mice, square symbols) dashed line, n = 6). Beginning at two months of age the animals were switched from a normal chow diet to the Paigen diet and then the food reservoir was weighed daily for each cage. **A** The apparent food intake per day per mouse was calculated as the difference between reservoir weights on sequential days. The per mouse values for the group housed, mixed genotypes are averages from all of the animals in each cage. **B** The apparent average food intake was determined over the 2–10 day period for each condition. There were no statistically significant differences.
(TIF)

Figure S3 **Relationships between plasma levels of oxytocin and corticosterone.** HypoE mice were housed in mixed genotype groups (HypoE and one or more SRBI^+/−^ApoeR61^h/h^ littermates per group, 4–5/cage, gray symbols, n = 15HypoE mice?) or singly (1/cage, black symbols, n = 17) Beginning at two months of age the animals were switched from a normal chow diet to the Paigen diet. After 19 days of Paigen diet feeding, the mice were weighed, plasma samples were harvested, and subsequently plasma levels of corticosterone and oxytocin were determined. Statistics were evaluated using Spearman’s rank correlation. The genotypes of mice were all HypoE, and group housed SR-BI^+/−^ApoeR61^h/h^ were not included in the analysis.
(TIF)
