# Supplementary figures and images for: Dietary Manipulation and Social Isolation Alter Disease Progression in a Murine Model of Coronary Heart Disease
Source: PLoS One. 2012 Oct 24;7(10):e47965. doi: 10.1371/journal.pone.0047965 (PMC3480446; doi:10.1371/journal.pone.0047965)

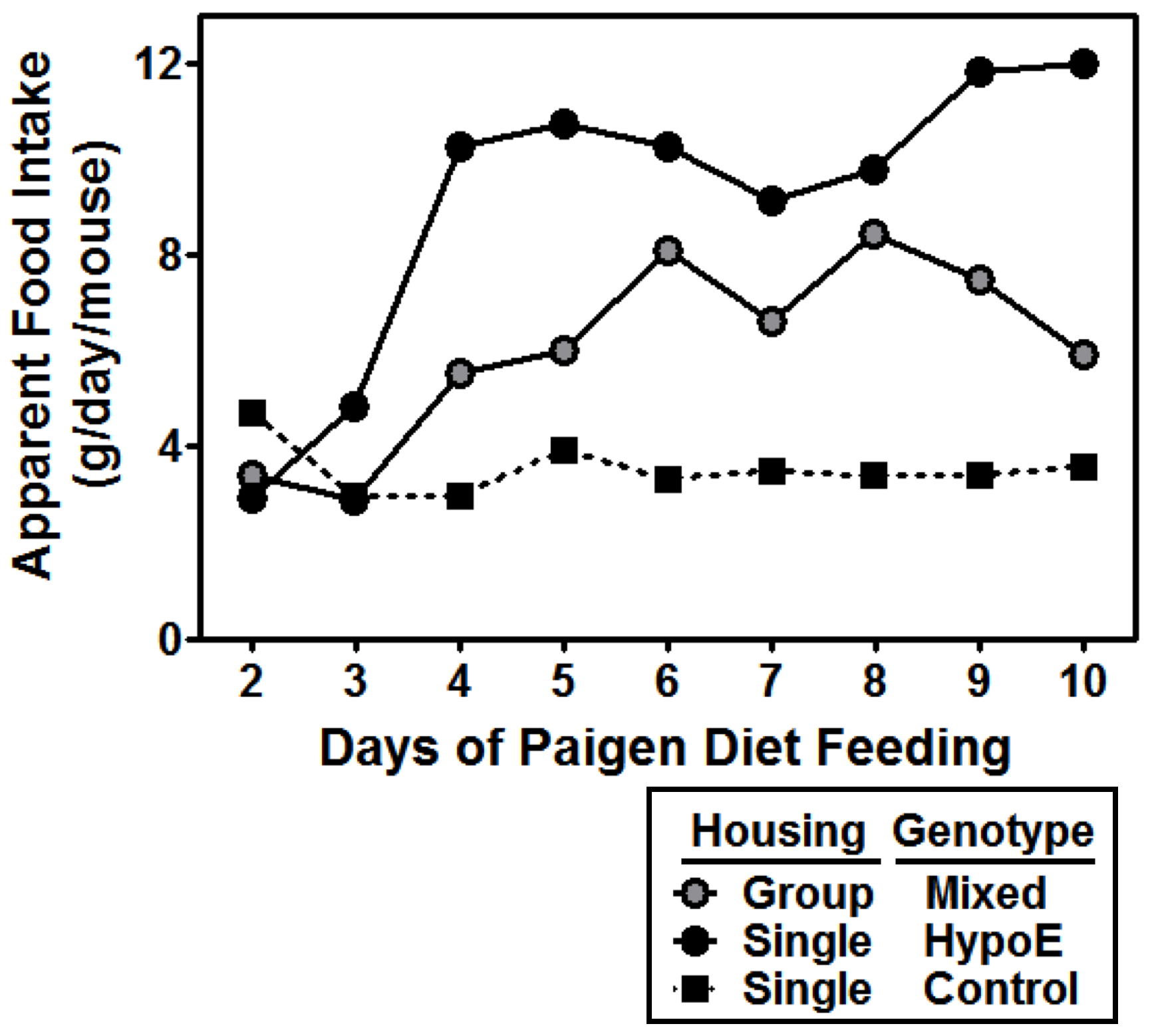

Supplement: Figure S1 — Effects of population density on apparent daily food intake of Paigen diet-fed HypoE female mice. Female mice were housed in mixed genotype groups (4 mice/cage, 2 HypoE and 2 SRBI+/−ApoeR61h/h littermates per group, 3 cages, gray symbols, n = 6 for HypoE mice) or singly (1/cage, black symbols; genotypes: HypoE, solid line, n = 5; wild-type (WT) or SRBI+/−ApoeR61h/h mice, dashed line, n = 4). Beginning at two months of age the animals were switched from a normal chow diet to the Paigen diet and then the food reservoir was weighed daily for each cage. The estimated food intake per day per mouse was calculated as the difference between reservoir weights on sequential days. The per mouse values for the group housed, mixed genotypes are averages from all of the animals in each cage. (TIF) [file pone.0047965.s001.tif]

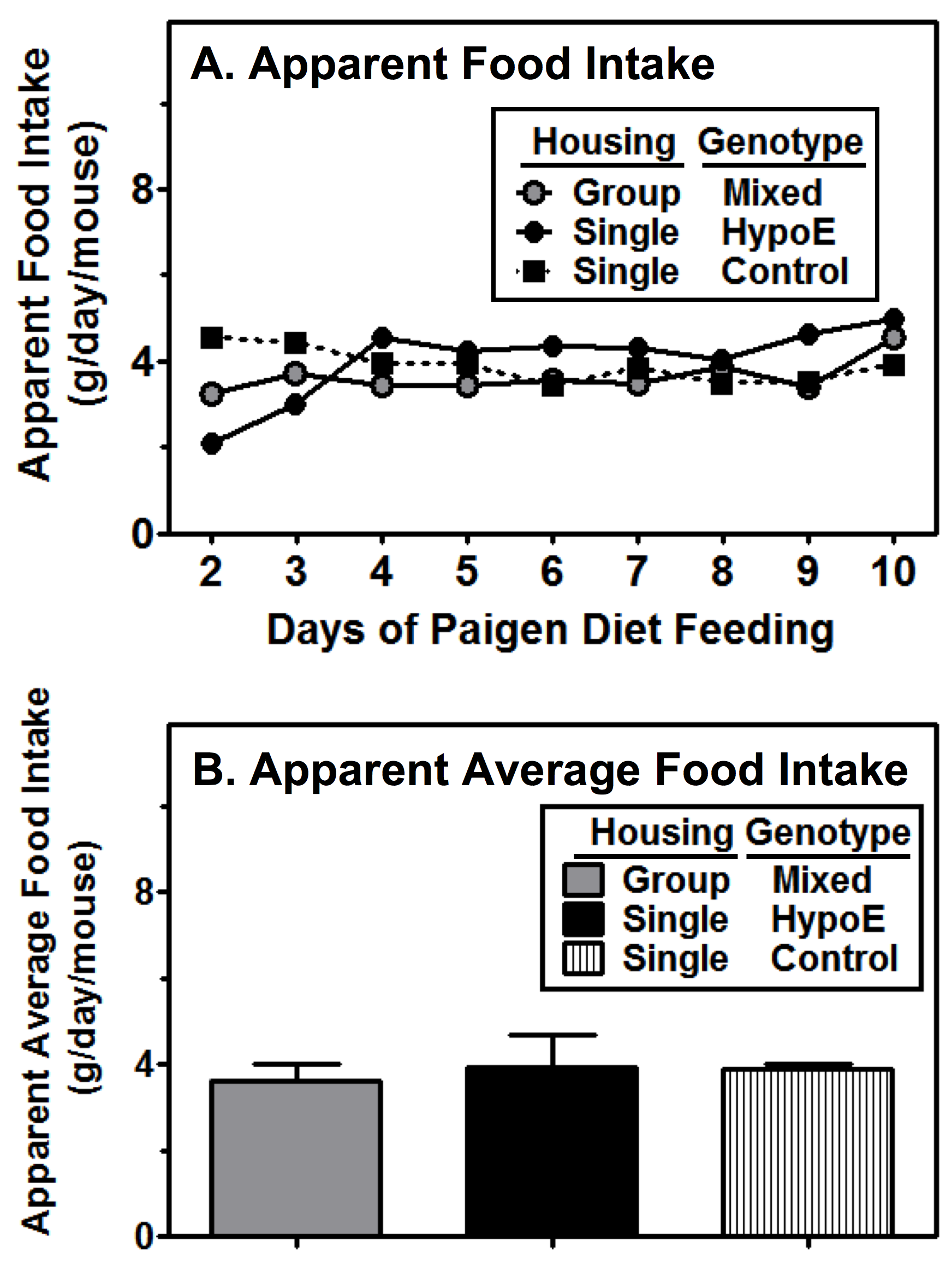

Supplement: Figure S2 — Effects of population density on apparent daily food intake of Paigen diet-fed HypoE male mice. Male mice were housed in mixed genotype groups (HypoE and one or more SRBI+/−ApoeR61h/h littermates per group, 3 cages, 4–5 mice/cage, 1–3 HypoE mice/cage, gray symbols, n = 6 for HypoE mice) or singly (1/cage, black symbols; genotypes: HypoE, solid line, n = 6; wild-type (WT) or SRBI+/−ApoeR61h/h mice, square symbols) dashed line, n = 6). Beginning at two months of age the animals were switched from a normal chow diet to the Paigen diet and then the food reservoir was weighed daily for each cage. A The apparent food intake per day per mouse was calculated as the difference between reservoir weights on sequential days. The per mouse values for the group housed, mixed genotypes are averages from all of the animals in each cage. B The apparent average food intake was determined over the 2–10 day period for each condition. There were no statistically significant differences. (TIF) [file pone.0047965.s002.tif]

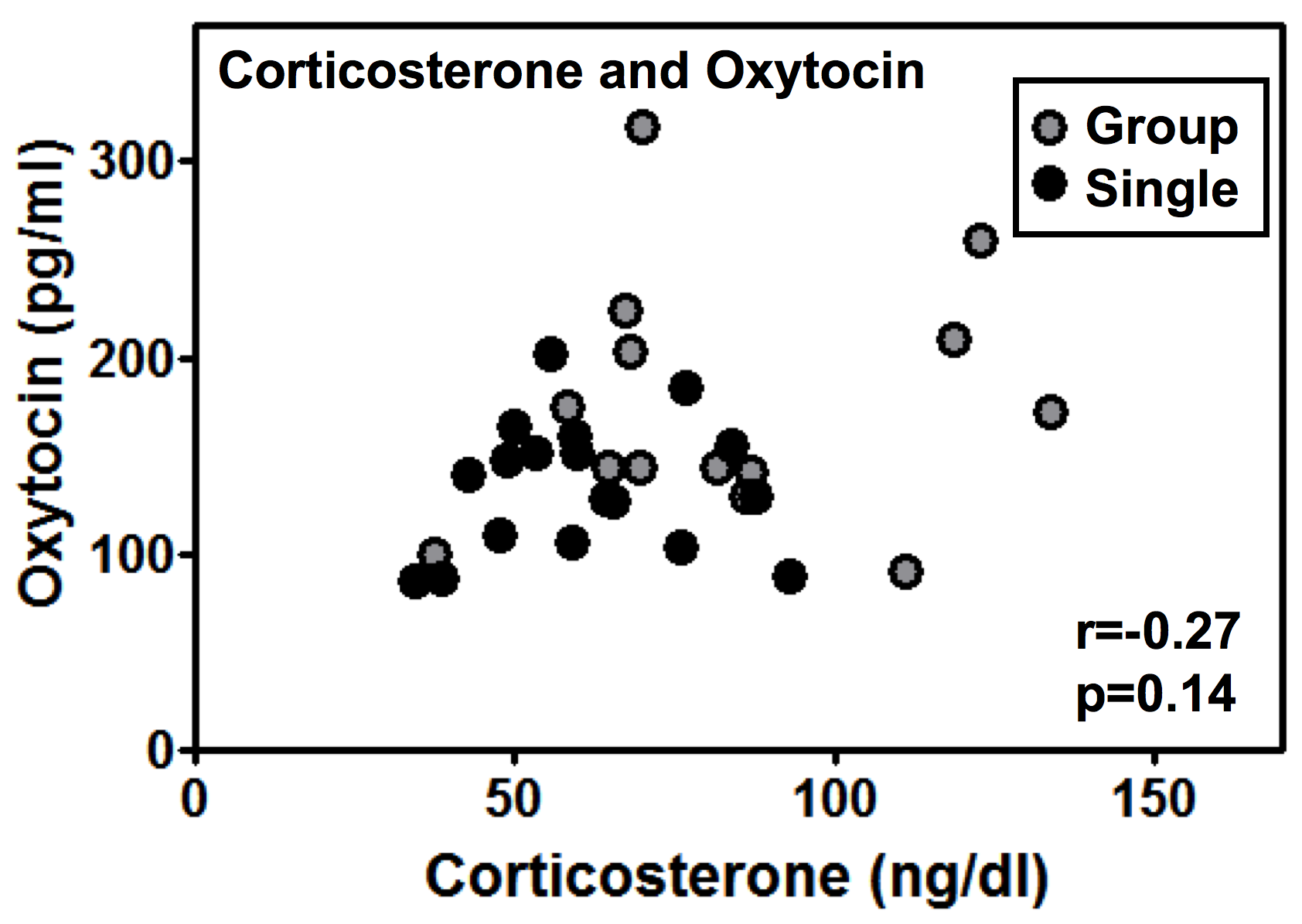

Supplement: Figure S3 — Relationships between plasma levels of oxytocin and corticosterone. HypoE mice were housed in mixed genotype groups (HypoE and one or more SRBI+/−ApoeR61h/h littermates per group, 4–5/cage, gray symbols, n = 15HypoE mice?) or singly (1/cage, black symbols, n = 17) Beginning at two months of age the animals were switched from a normal chow diet to the Paigen diet. After 19 days of Paigen diet feeding, the mice were weighed, plasma samples were harvested, and subsequently plasma levels of corticosterone and oxytocin were determined. Statistics were evaluated using Spearman’s rank correlation. The genotypes of mice were all HypoE, and group housed SR-BI+/−ApoeR61h/h were not included in the analysis. (TIF) [file pone.0047965.s003.tif]
